# The Large Uterus Classification System: a prospective observational study

**DOI:** 10.1111/1471-0528.16753

**Published:** 2021-06-08

**Authors:** S Uccella, RM Kho, S Garzon, J Casarin, PC Zorzato, F Ghezzi

**Affiliations:** ^1^ Division of Obstetrics and Gynaecology, Department of Maternal, Neonatal and Infant Health ASL Biella Biella Italy; ^2^ Department of Obstetrics and Gynaecology AOUI Verona, University of Verona Verona Italy; ^3^ Department Obstetrics and Gynecology Women's Health Institute, Cleveland Clinic Cleveland OH USA; ^4^ Department of Obstetrics and Gynaecology F. Del Ponte Hospital, University of Insubria Varese Italy

**Keywords:** Fibroids, laparoscopic hysterectomy, large uteri, Large Uterus Classification System

## Abstract

**Objective:**

To investigate the Large Uterus Classification System (LUCS) ability to predict surgical outcomes and complications in total laparoscopic hysterectomies (TLHs) for large uteri.

**Design:**

Prospective observational study.

**Setting:**

Two referral centres.

**Population or sample:**

Three hundred and ninety‐two women who underwent TLH for a large uterus (uterine fundus at or over the transverse umbilical line).

**Methods:**

Between 2004 and 2019, the intraoperative LUCS was estimated in all patients. The LUCS considers the uterine and adnexal vascular pedicles displacement. Type 1 is without vascular pedicles displacement. Type 2 has the cephalad displacement of adnexal vascular pedicles. The uterine vessels displacement regardless of adnexal pedicles defines Type 3.

**Main outcome measures:**

Patients’ characteristics with perioperative outcomes were prospectively collected and compared between the three types of large uteri.

**Results:**

Two hundred and fifty‐one (64%), 82 (20.9%) and 59 (15.1%) women had Type 1, Type 2 and Type 3 uteri, respectively. Women with Type 1 uteri had a lower uterine weight, shorter operative time, less blood loss and lower complication rates than women with Types 2 and 3. The conversion rate to laparotomy in Type 1 was similar to that in Type 2 (odds ratio [OR] 0.98; 95% CI 0.32–3.56) but lower than Type 3 (OR 0.35; 95% CI 0.14–0.97); in Type 2 it was lower than Type 3, although without the conventional statistical significance (OR 0.36; 95% CI 0.13–1.13; *P* = 0.07). Multivariable analysis showed that the uterine Type (1 versus 2–3) was independently associated with the total complications rate (OR 2.00; 95% CI 1.09–3.68; *P* = 0.02).

**Conclusions:**

The LUCS appears associated with surgical outcomes and complications, potentially stratifying the surgical risk and guiding the surgical technique in TLHs for large uteri.

**Tweetable abstract:**

The Large Uterus Classification System may predict outcomes in total laparoscopic hysterectomy of large uteri.

## Tweetable abstract

The Large Uterus Classification System may predict outcomes in total laparoscopic hysterectomy of large uteri.

## Introduction

Hysterectomy for benign conditions is one of the most common procedures in gynaecological practice. However, the technical difficulties of this intervention can be highly variable depending on the uterine size and possible concomitant conditions affecting the patient, such as pelvic adhesive disease, endometriosis, or presence and location of leiomyomas.^1^, ^2^, ^3^, ^4^

The removal of a large uterus invariably represents a surgical challenge, regardless of the surgical route – vaginal, open abdominal or endoscopic.^5^ Recent literature on this issue has shown, however, that the successful performance of a total laparoscopic hysterectomy (TLH) in the case of uteri weighing 1 kg or more is feasible in experienced hands and is associated with measurable patients benefits compared with traditional open surgery.^1^, ^6^, ^7^, ^8^ These advantages include a significantly lower rate of adverse events and severe complications (grade ≥2) according to the Clavien–Dindo classification.^1^, ^6^, ^7^, ^8^

Regardless of its potential advantages, TLH for large uteri remains a challenging procedure. The estimated weight alone does not appear to us enough to guide the surgical technique and predict surgical outcomes and complications. We observed that, apart from the actual weight of the uterus, a possible additional contributing factor, impacting the ability to perform a TLH for a large uterus, is the presence of displaced uterine or adnexal vascular pedicles. From this observation, we developed and propose the Large Uterus Classification System (LUCS), which is based on the intraoperative assessment of the uterine and adnexal vascular pedicles.

We hypothesised that if the surgical difficulty of a TLH is truly associated with the displacement of uterine or adnexal vessels, then the type of large uterus defined by this system may be associated with different surgical outcomes and complications. Therefore, the present study was conducted to evaluate whether our hypothesis is correct: the type of large uteri defined by the proposed LUCS is associated with the rate of conversion to open surgery, the operative time, the estimated blood loss, and total complications.

## Methods

### Study population, setting and methods

This is a prospective observational study. All consecutive patients with a large uterus (defined as having the uterine fundus at or over the transverse umbilical line) undergoing a TLH between June 2004 and September 2019 at the University of Insubria and the Hospital of Biella, Italy, were included. No age limit was set. Patients with absolute contraindication to laparoscopy and those with preoperative suspicion of a malignant uterine mass were excluded.

Preoperative, intraoperative and postoperative management of these patients is already described thoroughly elsewhere.^1^ Regarding the morcellation approach, most uteri were vaginally morcellated in an endobag. A minority of uteri were removed either by mini‐laparotomy or were fragmented using a laparoscopic scalpel with subsequent vaginal extraction.

All TLHs were performed by the same two surgeons: SU at the Hospital of Biella and the University of Insubria and FG at the University of Insubria. A detailed description of the initial laparoscopic inspection and appearance of the uterus was performed and documented in the operative notes of all patients. According to the proposed LUCS, the classification of the uterus was accomplished by the primary surgeon at the time of the initial laparoscopic inspection and was recorded in the operative reports. To ensure the correct attribution to the three pre‐specified categories, all surgical videos (operative notes when videos were not available) were reviewed by an independent internal evaluator who was not present at surgery (JC, PCZ). Possible disagreements between the original classification and the independent internal evaluator were resolved by an external independent reviewer (RMK). The external reviewer was masked regarding the classification accomplished by the primary surgeon and independent internal evaluators.

Data regarding patient demographics and clinical characteristics, operative time (minutes), estimated blood loss (ml), intraoperative and postoperative complications, conversion to open surgery, length of hospital stay, postoperative course and definitive uterine weight were recorded for each case. Complications were graded according to the Clavien–Dindo classification.^9^ Some of the patients included in the present series have been included in previous publications by our group.^1^, ^6^, ^7^, ^8^, ^10^, ^11^

The primary outcome was the total complication rate, defined as the sum of intraoperative and postoperative complication rates. Secondary outcomes were conversion to open surgery, operating time, estimated blood loss, length of hospital stay, and the rate of complications with a Clavien–Dindo classification score ≥2.^9^

Institutional Review Board approval was obtained. Written and informed consent was signed from all included patients. The present study protocol followed the Strengthening the Reporting of Observational Studies in Epidemiology (STROBE) statement.

### The Large Uterus Classification System

**Figure 1:**
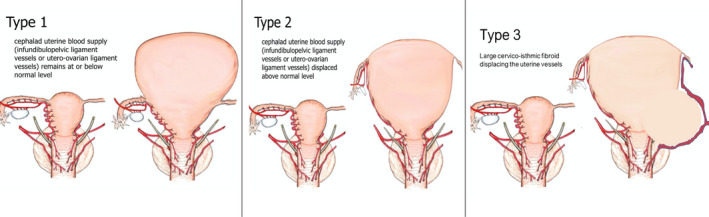
The Large Uterus Classification System. Type 1: large fundal fibroid(s), no cranial displacement of the vascular pedicles. Type 2: large fibroid(s) with the cranial displacement of the adnexal pedicles. Type 3: large cervico‐isthmic fibroid(s) with the displacement of the uterine vessels and the ureter.

Based on the hypothesised impact of displaced uterine and adnexal vascular pedicles on the ability to perform a TLH for large uteri, we developed the LUCS, which classifies each large uterus into one of three different types (Figure 1). The surgical technique adopted for each type of uterus is reported.

#### Type 1

Uterus with adnexal vascular pedicles (the infundibulopelvic ligament and utero‐ovarian ligaments) and uterine vessels that are approximately at the same level as a normal‐size uterus (Figure 1 – Type 1). This type of large uterus can be observed, for example, in the case of large fundal myoma. In this type of large uterus, we used steps for the TLH that resemble a standard procedure employed for normal‐sized uteri. Our technique has been previously described and published.^8^, ^10^, ^11^ Video S1 illustrates the surgical techniques for a normal‐size and an enlarged uterus weighing 1100 g.

#### Type 2

Uterus with cranially displaced adnexal vascular pedicles, at least on one side, but with uterine vessels approximately at the same level as a normal‐size uterus (Figure 1 – Type 2). In this type of large uterus, the technical difficulty involves managing the adnexal blood supply, which can be challenging to access using standard trocar positions. To overcome the technical challenges that this type of uterus entails, a 5‐mm 0‐degree scope can be inserted in one of the suprapubic ancillary trocars, and the umbilical trocar serves as an operative port. Alternatively, the optical and ancillary trocars were positioned more cephalad to optimise access to the displaced adnexal structures and their associated blood supply. Video S2 illustrates the technique used in a 3250‐g uterus with the cranial displacement of adnexa; the presence of extensive adhesions was due to two previous laparotomies.

#### Type 3

Uterus with displaced uterine vessels and with or without displaced adnexal vascular pedicles. This type of large uterus is characterised by markedly challenging access to the uterine artery and vein; the ureter can be displaced laterally or cranially. Usually, this condition is related to the presence of large cervical or lower uterine segment fibroids (Figure 1 – Type 3). For Type 3 uteri, we suggest the following possible surgical tips: (1) opening of the retroperitoneum with development of the pararectal and paravesical spaces to identify the ureters and to follow them to their entrance in the parametrial tunnel (this step allows the exposure of the ureter and prevention of inadvertent ureteral damage); (2) ligation of the uterine artery at its origin from the internal iliac artery (with the ureter under vision) to obtain devascularisation of the uterus at the beginning of the procedure and minimise possible bleeding (uterine vessels closure was performed using 5‐mm titanium clips, LIGAMAX™5 Endoscopic Multiple Clip Applier; Ethicon, Somerville, NJ, USA); (3) when present and feasible, isolation and cranial mobilisation of the cervical fibroid to obtain better upward mobilisation of the uterus and avoid excessive bleeding when in close proximity to the deep uterine vein, (4) completion of the procedure after obtaining adequate mobilisation of the uterus. Video S3 illustrates the case of a 1200‐g uterus with an 8‐cm left cervical fibroid and a 4‐cm right cervical fibroid extending bilaterally to the parametria and displacing the cervix. The positioning of the uterine manipulator was not successful; therefore, we used a sponge inserted transvaginally to identify the vaginal fornix.

### Statistical analysis

Conventional descriptive statistics were used to report patients’ demographic and clinical characteristics, intraoperative and postoperative complications, surgical data, and perioperative outcomes. Continuous variables were expressed as mean and standard deviation or as median and interquartile range based on the distribution. Normality was tested with the D'Agostino & Pearson omnibus normality test. When the corresponding *P* value was <0.10, normality distribution was rejected. Categorical variables were expressed as frequency and percentage. Analysis of variance and Kruskal–Wallis tests were used to compare continuous and ordinal variables between the three uterine types as appropriate. Chi‐square test and Fisher’s exact test were used to compare categorical data. The post hoc pairwise comparisons of Type 1 versus Type 2, Type 2 versus Type 3 and Type 1 versus Type 3 were performed with independent *t* test, Wilcoxon rank‐sum test, chi‐square test and Fisher’s exact test as appropriate. *P* values were adjusted for multiple comparisons with the Bonferroni method. Statistical significance was defined when *P* < 0.05 (two‐tailed).

Multiple logistic regression analysis was performed to identify factors associated with the primary outcome. The multivariable model was built using the hierarchical method. Uterine weight and type (Type 2–3 versus Type 1), age and previous abdominal surgery were chosen as possible independent predictors of total complications. Continuous variables (uterine weight and age) were introduced in the model as binary variables; the ROC curves were used to identify the optimal cut‐off value to change the variable type (the one with the best specificity and sensitivity for total complications).

Statistical analysis was performed using SPSS version 22 (IBM Corp., Armonk, NY, USA).

## Results

A total of 392 patients having a uterus with uterine fundus at or over the transverse umbilical line underwent TLH during the study period. The procedure was completed laparoscopically in 363 cases (92.6%). We classified 251 (64%) patients as Type 1 uterus, 82 (20.9%) women as Type 2, and 59 (15.1%) as Type 3 uterus. The uterine type assignment was not changed by the independent internal evaluators, who confirmed all assigned uterine types.

Patients’ clinical and demographic characteristics and study outcomes are reported in Table 1, stratified by uterine type, according to the LUCS. The three groups were comparable in terms of demographic characteristics, reporting similar age, body mass index, and parity between the three types. Conversely, the three groups had statistically significantly different clinical characteristics and study outcomes. Uterine weight was lower in Type 1 than Type 2 and 3 (1124 g versus 1418 g for Type 2 and 1307 g for Type 3, *P* < 0.001). Significant differences in terms of conversions to open surgery (6% in Type 1, 6.1% in Type 2 and 15.3% in Type 3; *P* = 0.03), operative time (112, 137,and 147 minutes for Type 1, 2 and 3 uteri, respectively; *P* < 0.001), blood loss (210, 342, 338 ml for Type 1, 2 and 3 uteri, respectively; *P* < 0.001), postoperative complications (8.8, 14.6, and 18.6% for Type 1, 2 and 3 uteri, respectively; *P* = 0.04) and total complications (9.6, 17, and 22% for Type 1, 2 and 3 uteri, respectively; *P* = 0.018) were noted among groups. The hospital stay was similar across the three different uterine types (1.9, 2, and 2.2 days in Type 1, 2 and 3 uteri, respectively; *P* = 0.32).

**Table 1:** Comparison of patients' clinical and demographic characteristics and study outcomes between the three groups

	Type 1	Type 2	Type 3	*P* value
Number of cases	251 (64%)	82 (20.9%)	59 (15.1%)	–
Age (years)	47.7 (32–66)	48.9 (36–75)	47.7 (37–68)	0.33
Body mass index (kg/m^2^)	25.1 (18.6–35)	27.2 (18.3–36.6)	25 (20–41)	0.54
Nulliparous	102 (40.6%)	39 (47.6%)	26 (44.1%)	0.53
Uterine weight (g)	1124 ± 410	1418 ± 622	1307 ± 452	<0.001
Conversion to open surgery	15/251 (6%)	5/82 (6.1%)	9/59 (15.3%)	0.03
Adnexectomy				
Bilateral	81/251 (32.3%)	30/82 (36.6%)	17/59 (28.8%)	0.61
Monolateral	5/251 (1.9%)	4/82 (4.9%)	1/59 (1.7%)	0.32
Unplanned adnexectomy	0/251	0/82	0/59	1
Operative time (minutes)	112 ± 56	137 ± 49	147 ± 58	<0.001
Blood loss (ml)	210 ± 253	342 ± 450	338 ± 257	<0.001
Intraoperative complications	2 (0.8%)	2 (2.4%)	2 (3.4%)	0.26
Postoperative complications	22 (8.8%)	12 (14.6%)	11 (18.6%)	0.04
Organ injuries				
Bladder lesions	1	0	1	>0.99
Ureteral complications	0	0	1	
Bowel lesions	1	0	0	
Total complications	24 (9.6%)	14 (17%)	13 (22%)	0.018
Postoperative complications ≥ Grade 2	13 (5.2%)	4 (4.9%)	4 (6.8%)	0.87
Hospital stay (days)	1.9 ± 1.3	2 ± 1.4	2.2 ± 1.3	0.32
Malignancy in the final pathology	0/251	0/82	0/59	1

Values have been reported as absolute number and percentage (%) for dichotomous variables and mean ± standard deviation for continuous variables. Postoperative complications have been graded according to the Clavien–Dindo classification.

A total of 4 (1%) organ injuries occurred: two bladder lesions (one in Type 1 and one in Type 3 uteri), which were immediately recognised and repaired laparoscopically without sequelae: one serosal lesion of the sigmoid (in a Type 1 uterus), which was sutured laparoscopically with no consequence, and one ureteric fistula (in a Type 3 uterus), which was diagnosed 10 days postoperatively, repaired and treated with placement of a ureteral J‐J stent for 2 months. The rate of Clavien–Dindo grade ≥2 postoperative complications was similar among groups. Regarding grade ≥2 postoperative complications, there were nine cases of infected postoperative pelvic collections (six in Type 1 uteri and three in Type 3 uteri), three cases of vaginal cuff dehiscence (two in Type 1 uteri and one in a Type 2 uterus), three cases of vaginal bleeding requiring readmission (all in Type 1 uteri), one case of haemoperitoneum requiring reintervention (in a patient with Type 1 uterus), one case of ureteral stricture successfully treated with stent (Type 3 uterus), three cases of postoperative fever treated with intravenous antibiotics (one1 in Type 1 and two in Type 2 uteri), and one postoperative pneumonia (Type 2 uterus).

The results of the post hoc pairwise comparison between Type 1 and Type 2 uteri are provided in Table S1. A longer operative time and a higher blood loss were noted in Type 2 versus Type 1 uteri. A higher rate of total complications was registered in Type 2 versus Type 1 uteri, although the total complication rate did not reach the conventional level of statistical significance (17% versus 9.6%, respectively; *P* = 0.06; odds ratio [OR] 1.95, 95% CI 0.97–3.9). On the other hand, the rates of conversion to open surgery were similar (6.1% versus 6%; *P* = 1.00; OR 1.02, 95% CI 0.40–2.68) between these two groups.

The post hoc pairwise comparison between Type 1 and Type 3 uteri is shown in Table S2. The operative time, blood loss, rate of postoperative complications, total complications and conversion rate were significantly higher in Type 3 versus Type 1 uteri.

Table S3 shows the post hoc pairwise comparison between Type 2 and Type 3 uteri. The two categories were similar in terms of operative outcomes, but there was a higher rate of conversion to open surgery in the group of Type 3 than Type 2 uteri, although without the conventional level of statistical significance (6.1% versus 15.3%; *P* = 0.07; OR 2.77, 95% CI 0.91–7.68).

At the multivariable analysis (Table 2), the classification as Type 2–3 uteri was the only independent predictor of a higher rate of total complications than Type 1 uteri (OR 2.00, 95% CI 1.09–3.68; *P* = 0.02). Uterine weight was associated with the rate of total complications at univariate analysis (*P* = 0.03), but it was not independently associated with the primary outcome at multivariable analysis (OR 1.64, 95% CI 0.81–3.31; *P* = 0.17). Neither age nor previous abdominal surgery was a predictor of total complications.

**Table 2:** Multivariable analysis of factors associated with total complications of laparoscopic hysterectomy for large uteri

Factor	Univariate analysis			Multivariable analysis	
	Complication (*n* = 51)	No complication (*n* = 341)	*P* value	OR (95% CI)	*P* value
Age (years)	47.5 (39–68)	48 (32–75)	0.94	0.80 (0.42–1.63)	0.50
Uterine weight (g)	1329 ± 509	1196 ± 477	0.03	1.64 (0.81–3.31)	0.17
Type 2–3 large uterus	27 (52.9%)	114 (32.9%)	0.007	2.00 (1.09–3.68)	0.02
Previous abdominal surgery	3 (5.8%)	41 (12%)	0.24	0.48 (0.14–1.49)	0.24

Age and uterus weight were introduced in the multivariable model as binary variables, the optimal cut‐off value to convert variables in binary type was calculated using receiver operating characteristics curves.

## Discussion

### Main findings

We observed that, in the case of a large uterus having its fundus at or over the transverse umbilical line, the uterine type defined according to the proposed LUCS was associated with surgical outcomes regardless of the uterine weight. Specifically, the LUCS uterine type was associated with different conversion rates, operative time, blood loss and total complications.

### Interpretation

When conducted systematically, the intraoperative use of the LUCS system may be a useful tool to classify large uteri at the beginning of the TLH and predict surgical outcomes and complications. Although a preoperative stratification according to LUCS, using ultrasound and magnetic resonance imaging (MRI), is currently under investigation and yet to be validated, the results of the present study provide adequate background to promote further research in this field. The adoption of the LUCS may allow the surgical team to tailor the operative technique to the difficulty of the case to optimise surgical outcomes.

Existing studies evaluating hysterectomy outcomes in large uteri do not consider the location and relationship to the vascular pedicles.^5^ Our analysis highlights the impact of these contributing factors on surgical outcomes. Compared with the other uterine types, the Type 1 uterus, approached using the standard TLH technique, was associated with less blood loss, shorter operative time, fewer complications, and fewer conversions to open surgery. Similarly, when hysterectomy outcomes for Type 2 and Type 3 uteri were compared, there was a higher conversion rate to laparotomy in Type 3 versus Type 2 uteri, although without the conventional level of statistical significance. These findings support the hypothesis that the type of large uterus defined by the proposed LUCS is associated with different surgical outcomes of the TLH.

However, most relevant differences in primary and secondary outcomes were observed between Type 1 versus Type 2 and 3 uteri, which reported, at the same time, a statistically significant lower uterine weight among Type 1 uteri compared with the other uterine types. Nevertheless, the multivariable model confirmed that the uterine type defined by the LUCS is an independent predictor of total complications rate regardless of the observed associated uterine weight. The multivariable model supports our observation that the presence of displaced uterine and adnexal vascular pedicles is associated with the overall surgical complexity of the operation regardless of uterine weight.

**Figure 2:**
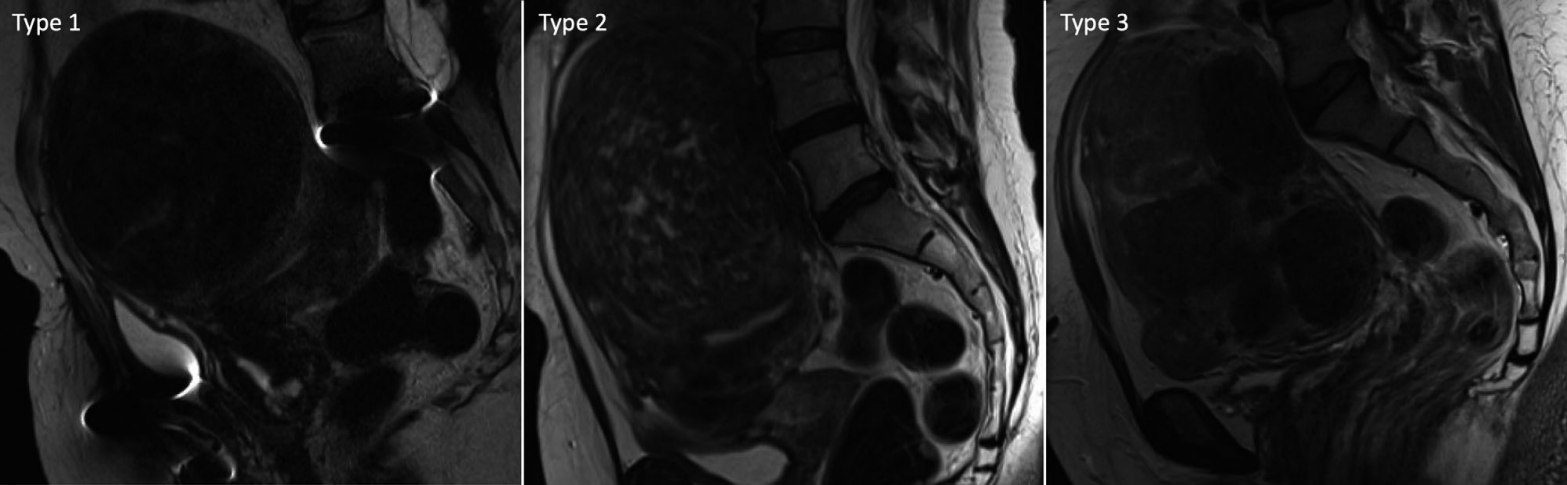
Possible examples of the Large Uterus Classification System preoperative appearance at the magnetic resonance imaging. Type 1: large uterus with voluminous anterior‐fundal fibroid. Anatomic insertions of adnexal vascular pedicles and uterine vessels are approximately at the same level as a normal‐size uterus. Type 2: large uterus with a voluminous antero‐fundal fibroid that cranially displaced the adnexal vascular pedicles insertion. Type 3: large uterus with multiple voluminous fibroids that cranially displaced the adnexal vascular pedicles insertion and distorted the pelvic anatomy with the displacement of uterine vessels.

At present, we are in the process of identifying a preoperative imaging protocol that could adequately characterise the type of large uterus and its uterine and adnexal vascular supply. Our proposed LUCS, based on an intraoperative evaluation, demonstrated a correlation with worse surgical outcomes that increase from type 1 to type 3 large uteri. The adoption of ultrasound with colour Doppler and possibly MRI with angiography, currently used to identify complex uterine arteriovenous malformations and intravenous leiomyomatosis,^12^ would probably be helpful for preoperative LUCS classification (Figure 2). Collaboration with imaging experts to develop a structured preoperative imaging protocol with MRI or ultrasound would be the next logical step to expand further the utility of the LUCS. In this regard, other elements identifiable on preoperative imaging, such as hydroureter and hydronephrosis, may be considered as additional risk factors or markers of displaced uterine or adnexal vascular pedicles. In gynaecological surgery, hydronephrosis grade ≥2 has been associated with worse outcomes.^13^

Multiple studies have described the feasibility of TLH in women with large uteri.^1^, ^3^, ^6^, ^7^, ^8^, ^14^, ^15^, ^16^, ^17^, ^18^, ^19^, ^20^ Several articles suggest that the ability to remove a large uterus laparoscopically depends primarily on the surgeon’s proficiency.^2^, ^4^ Although it is commonly accepted that the completion of the procedure in a minimally invasive manner provides significant benefits to the woman in terms of perioperative complications,^1^ there is no study evaluating a systematic categorisation of the large uterus for the prediction of surgical difficulty and outcomes. Our proposal for a simple classification, albeit conducted intraoperatively at this time, may represent an effective method to stratify cases and plan the operative strategy given the anticipated surgical difficulty, such as the preparation of blood products or request for additional surgical assistance.

### Strengths and limitations

The strengths of the present study include the systematic and prospective assignment of the LUCS in a large sample size of TLHs performed for markedly enlarged uteri, the involvement of two large referral centres and the systematic analysis of surgical outcomes in relation to the LUCS with prospective data collection. To limit misclassification, all available surgical videos or operative reports were reviewed independently.

Nevertheless, caution is required. The main limitation is that the proposed LUCS was not validated in an external cohort of patients by other groups of surgeons in a different context. This is essential to appropriately assess the reproducibility and validity of the LUCS and, mainly, to discover whether surgeons with different levels of experience could reproduce results. Surgical expertise is a well‐known confounding factor in surgical research,^21^ and results could have been influenced by the surgical expertise of the authors. However, concerning this point, on the one hand, surgeon experience may affect the ability to classify uteri; on the other hand, surgeon experience usually attenuates differences in complication rates and may mask some additional utility of the classification.^21^ In any case, any proposed classification should be reviewed by a panel of internal and external experts and approved by an established scientific organisation or specialist Society with a standardised international consensus before being widely used. All these limitations highlight the need for further studies to confirm the reproducibility and validity of the LUCS. Specifically, it should be verified whether our findings may be generalisable to lower‐volume centres with less laparoscopic expertise. Finally, we need to confirm that the LUCS works when the TLH is robotically assisted. A prospective multicentre study for external validation of this proposed classification system would be helpful. The development of highly accurate, cost‐effective and accessible preoperative imaging for the proposed LUCS would also be warranted to avoid the possible morbidity associated with a change of surgical approach intraoperatively.

## Conclusion

Our findings from this prospective observational study suggest that the displacement of the vascular pedicles correlates with the surgical outcomes and complications, representing a potential marker of technical difficulties. The proposed LUCS classification may represent a simple intraoperative method to deliver valuable information to the surgeon regarding surgical outcomes and the risk of complications in TLHs for enlarged uteri. However, external validation of the LUCS is needed before achieving a definitive conclusion on its utility. Other surgical groups are encouraged to validate the usefulness of this proposed classification. Moreover, further research should focus on developing an accurate preoperative method to assess the uterine type before surgery to plan the surgical strategy preoperatively. This would improve surgical outcomes, avoiding the possible morbidity associated with a change of surgical approach intraoperatively.

### Disclosure of interests

The authors have no proprietary, financial, professional, or other personal interest of any nature in any product, service, or company to disclose. The authors alone are responsible for the content and writing of the paper. Completed disclosure of interests form available to view online as supporting information.

### Contribution to authorship

All the authors conform to the International Committee of Medical Journal Editors (ICMJE) criteria for authorship, contributed to the intellectual content of the study and gave approval for the final version of the article. SU and FG conceptualised the study. SU, FG and RMK designed the study. SU and FG performed laparoscopic total hysterectomies. JC and PCZ prospectively collected data. SU, SG and PCZ managed the data set and performed statistical analyses. SU, RMK and SG wrote the manuscript. All authors contributed to the interpretation of the results, as well as to the writing and editing of the manuscript.

### Details of ethics approval

All the design, analysis, interpretation of data, drafting and revisions conform to the Helsinki Declaration, the Committee on Publication Ethics (COPE) guidelines (http://publicationethics.org/), and the STROBE (Strengthening the Reporting of Observational Studies in Epidemiology) statement, available through the EQUATOR (enhancing the quality and transparency of health research) network (https://www.equator‐network.org). The study was approved by the Institutional Review Boards of Biella and Varese (PROT. 03/2004; 3 February 2004). All women gave consent for study participation and anonymised data collection and analysis for research purposes.

### Funding

The study was not funded.

## Supporting information

**Table S1.** Post hoc pairwise comparison between Type 1 and Type 2 uteri.
**Table S2.** Post hoc pairwise comparison between Type 1 and Type 3 uteri.
**Table S3.** Post hoc pairwise comparison between Type 2 and Type 3 uteri.

**Video S1.** Comparison between total laparoscopic hysterectomy for normal sized uterus (left) and Type 1 Large Uterus (right).

**Video S2.** Total laparoscopic hysterectomy for Type 2 Large Uterus.

**Video S3.** Total laparoscopic hysterectomy for Type 3 Large Uterus.

Supplementary Material

Supplementary Material

Supplementary Material

Supplementary Material

Supplementary Material

Supplementary Material

## Data Availability

Data available on request from the authors.
