# Supplementary material for: The Large Uterus Classification System: a prospective observational study
Source: BJOG. 2021 Jun 8;128(9):1526–33. doi: 10.1111/1471-0528.16753 (PMC8362220; doi:10.1111/1471-0528.16753)
Supplement: Supplementary file 1 — Table S1. Post hoc pairwise comparison between Type 1 and Type 2 uteri. Table S2. Post hoc pairwise comparison between Type 1 and Type 3 uteri. Table S3. Post hoc pairwise comparison between Type 2 and Type 3 uteri. [file BJO-128-1526-s007.docx]

| **Table S1.** Posthoc pair-wise comparison between Type 1 and Type 2 uteri. | | | |
| --- | --- | --- | --- |
|  | **Type 1** | **Type 2** | **P value** |
| Number of cases | 251 | 82 | - |
| Uterine weight (grams) | 1124 ± 410 | 1418 ± 622 | <0.001 |
| Conversion to laparotomy | 15/251 (6%) | 5/82 (6.1%) | 1.00 |
| Operative Time (min) | 112 ± 56 | 137 ± 49 | <0.001 |
| Blood Loss (ml) | 210 ± 253 | 342 ± 450 | 0.001 |
| Intraoperative complications | 2 (0.8%) | 2 (2.4%) | 0.24 |
| Postoperative complications | 22 (8.8%) | 12 (14.6%) | 0.13 |
| Total Complications | 24 (9.6%) | 14 (17%) | 0.06 |
| Post-operative complications > Grade2 | 13 (5.2%) | 4 (4.9%) | 0.91 |
| Hospital stay (days) | 1.9 ± 1.3 | 2 ± 1.4 | 0.53 |

Values have been reported as absolute number and percentage (%) for dichotomous variables, while mean +/- standard deviation for continuous variables. BMI= Body Mass Index.

Postoperative complications have been graded according to the Clavien-Dindo classification.

| **Table S2.** Posthoc pair-wise comparison between Type 1 and Type 3 uteri. | | | |
| --- | --- | --- | --- |
|  | **Type 1** | **Type 3** | **P value** |
| Number of cases | 251 (64%) | 59 (15.1%) | - |
| Uterine weight (grams) | 1124 ± 410 | 1307 ± 452 | 0.006 |
| Conversion to open surgery | 15 (6%) | 9 (15.3%) | 0.02 |
| Operative time (min) | 112 ± 56 | 147 ± 58 | <0.001 |
| Blood loss (ml) | 210 ± 253 | 338 ± 257 | <0.001 |
| Intraoperative complications | 2 (0.8%) | 2 (3.4%) | 0.11 |
| Postoperative complications | 22 (8.8%) | 11 (18.6%) | 0.01 |
| Total complications | 24 (9.6%) | 13 (22%) | 0.007 |
| Post-operative complications > Grade2 | 13 (5.2%) | 4 (6.8%) | 0.63 |
| Hospital stay (days) | 1.9 ± 1.3 | 2.2 ± 1.3 | 0.13 |

Values have been reported as absolute number and percentage (%) for dichotomous variables, while mean +/- standard deviation for continuous variables. Postoperative complications have been graded according to the Clavien-Dindo classification.

| **Table S3.** Posthoc pair-wise comparison between Type 2 and Type 3 uteri. | | | |
| --- | --- | --- | --- |
|  | **Type 2** | **Type 3** | **P value** |
| Number of cases | 82 (20.9%) | 59 (15.1%) | - |
| Uterine weight (grams) | 1418 ± 622 | 1307 ± 452 | 0.24 |
| Conversions | 5/82 (6.1%) | 9/59 (15.3%) | 0.07 |
| Operative time (min) | 137 ± 49 | 147 ± 58 | 0.26 |
| Blood loss (ml) | 342 ± 450 | 338 ± 257 | 0.89 |
| Intraoperative complications | 2 (2.4%) | 2 (3.4%) | 0.74 |
| Postoperative complications | 12 (14.6%) | 11 (18.6%) | 0.52 |
| Total complications | 14 (17%) | 13 (22%) | 0.46 |
| Post-operative complications > Grade2 | 4 (4.9%) | 4 (6.8%) | 0.63 |
| Hospital stay (days) | 2 ± 1.4 | 2.2 ± 1.3 | 0.46 |

Values have been reported as absolute number and percentage (%) for dichotomous variables, while as mean +/- standard deviation for continuous variables.

Postoperative complications have been graded according the Clavien-Dindo classification.
